# A window into the future? MRI for evaluation of neuromyelitis optica spectrum disorder throughout the disease course

**DOI:** 10.1177/17562864211014389

**Published:** 2021-05-09

**Authors:** Jacqueline M. Solomon, Friedemann Paul, Claudia Chien, Jiwon Oh, Dalia L. Rotstein

**Affiliations:** University of Toronto, Department of Medicine, Toronto, ON, Canada; St. Michael’s Hospital, Toronto, ON, Canada; Experimental and Clinical Research Center, Max Delbrueck Center for Molecular Medicine and Charité Universitaetsmedizin Berlin, Berlin, Germany; NeuroCure Clinical Research Center, Charité Universitaetsmedizin Berlin, corporate member of Freie Universität Berlin, Humboldt-Universität zu Berlin, and Berlin Institute of Health, Berlin, Germany; Experimental and Clinical Research Center, Max Delbrueck Center for Molecular Medicine and Charité Universitaetsmedizin Berlin, Berlin, Germany; NeuroCure Clinical Research Center, Charité Universitaetsmedizin Berlin, corporate member of Freie Universität Berlin, Humboldt-Universität zu Berlin, and Berlin Institute of Health, Berlin, Germany; Department of Psychiatry and Psychotherapy, Charité Universitaetsmedizin Berlin, corporate member of Freie Universität Berlin, Humboldt-Universität zu Berlin, and Berlin Institute of Health, Berlin, Germany; University of Toronto, Department of Medicine, Toronto, ON, Canada; St. Michael’s Hospital, Toronto, ON, Canada; St. Michael’s Hospital, 30 Bond Street, Shuter 3-018, Toronto, ON, M5B 1W8, Canada

**Keywords:** disease activity, MRI, NMOSD, prognosis

## Abstract

Neuromyelitis optica spectrum disorder (NMOSD) is a relapsing, inflammatory disease of the central nervous system marked by relapses often associated with poor recovery and long-term disability. Magnetic resonance imaging (MRI) is recognized as an important tool for timely diagnosis of NMOSD as, in combination with serologic testing, it aids in distinguishing NMOSD from possible mimics. Although the role of MRI for disease monitoring after diagnosis is not as well established, MRI may provide important prognostic information and help differentiate between relapses and pseudorelapses. Increasing evidence of subclinical disease activity and the emergence of newly approved, highly effective immunotherapies for NMOSD adjure us to re-evaluate MRI as a tool to guide optimal treatment selection and escalation throughout the disease course. In this article we review the role of MRI in NMOSD diagnosis, prognostication, disease monitoring, and treatment selection.

## Introduction

**Figure 1.**
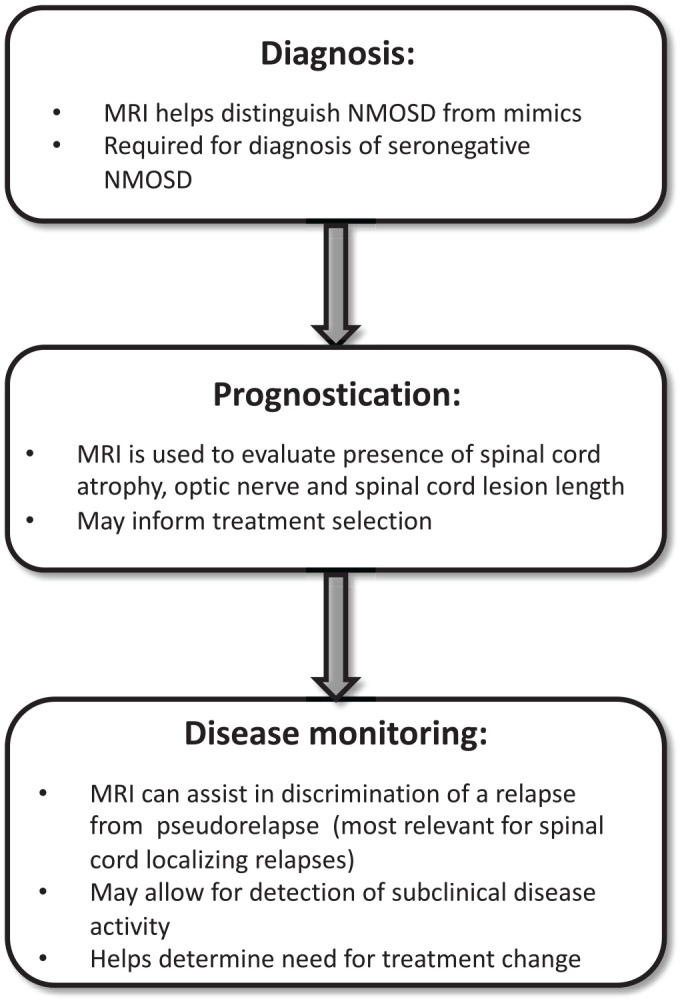
The use of magnetic resonance imaging (MRI) for three key phases during the disease course of neuromyelitis optica spectrum disorder (NMOSD): diagnosis, prognostication, and disease monitoring.

Neuromyelitis optica spectrum disorder (NMOSD) is a relapsing inflammatory disease of the central nervous system characterized by disabling optic neuritis, longitudinally extensive transverse myelitis (LETM), and brain/brainstem attacks. Initially thought to be a rare and severe variant of multiple sclerosis (MS), it has become apparent that NMOSD is a pathologically distinct entity associated with serum antibodies to the astrocyte water channel aquaporin-4 (AQP4) in the majority of patients.^1–4^ Likewise, the recent discovery of myelin oligodendrocyte glycoprotein antibodies (MOG-IgG) has led to the detection of MOG-IgG in a portion of AQP4-IgG seronegative NMOSD patients. MOG antibody disease (MOGAD) has therefore emerged as a separate disease entity with a distinct pathogenesis from classic NMOSD.^5,6^ The relevance of magnetic resonance imaging (MRI) in NMOSD has been clearly established for diagnosis, but not for long-term monitoring, in contrast to MS, for which MRI is routinely employed to monitor disease activity and treatment response.^7^ This may stem from the traditional view that long-term disability in NMOSD arises only from clinical relapses, and thus routine imaging is unlikely to change management. However, with the emergence of highly effective immunotherapies for NMOSD and growing evidence of subclinical disease activity, it is imperative to highlight the potential utility of MRI at various disease stages in NMOSD (Figure 1).^8–17^ In fact, MRI may prove to be a key tool for diagnostic accuracy and personalized therapy at the earliest possible juncture of the disease course. First, MRI facilitates diagnosis, with differentiation of NMOSD from MS, MOGAD, and other mimics in combination with serologic testing for AQP4 and MOG antibodies, preferably by employing cell-based assays.^18,19^ Second, MRI findings such as spinal cord atrophy, and spinal cord and optic nerve lesion length, may provide prognostic information about NMOSD disease course^20–24^ to help direct optimal therapy including institution of newer high-efficacy therapies. Third, MRI may assist with disease monitoring to distinguish relapses from pseudorelapses, which has important therapeutic implications. Lastly, MRI, including advanced MRI techniques, may flag subclinical disease activity. The predictive value of subclinical disease activity for future relapses and long-term disability in NMOSD requires further study but, if confirmed, could allow for pre-emptive escalation in therapy.

## Diagnosis

MRI is a critical diagnostic tool when evaluating patients with a clinical presentation suspicious for NMOSD, not only to confirm the diagnosis, but also to exclude possible mimics with similar clinical manifestations. For seronegative NMOSD, MRI is particularly important, given the increased diagnostic uncertainty. In these patients, supportive MRI features are required to fulfill the more stringent diagnostic criteria, which include specific clinical characteristics with associated neuroimaging findings.^25,26^

Although brain MRI in NMOSD patients is often unremarkable, lesions are detected 50–85% of the time in patients fulfilling the revised 2006 NMO diagnostic criteria, with brain abnormalities found in 43–70% of patients at initial presentation.^25,27–30^ The most common abnormalities on MRI of the brain in NMOSD are non-specific punctate hyperintensities in the subcortical and deep white matter on T2-weighted or fluid-attenuated inversion recovery (FLAIR) sequences.^28^ However, lesions characteristic of NMOSD may be present and can be useful in distinguishing NMOSD from MS, MOGAD, and other inflammatory and non-inflammatory conditions.

### Brain lesions

Characteristic brain lesions in NMOSD are typically periependymal, located around the cerebral aqueduct and the third and fourth ventricles in circumventricular organs that are highly vascularized and devoid of a blood–brain barrier.^28^ These include lesions in the diencephalon (hypothalamus and thalamus) and brainstem, both areas found to have high AQP4 IgG expression.^31^ Periaqueductal lesions, in particular, can be seen in NMOSD, as well as MOGAD, but are very rare in MS.^32,33^ Lesions in the emesis-inducing center, area postrema, of the dorsal medulla are common and characteristic of NMOSD, and may be responsible for the initial attack with symptoms of nausea, vomiting, and intractable hiccups.^34,35^ These lesions may be transient, therefore it is important to image with MRI soon after symptom onset; gadolinium-enhanced sequences may provide added sensitivity for detection.^33,34^

Other brain MRI abnormalities observed in NMOSD are large (>3 cm) and confluent cerebral hemispheric lesions located in the subcortical white matter.^36^ These lesions may have a tumefactive appearance or may resemble lesions typical of acute disseminated encephalomyelitis (ADEM) and posterior reversible encephalopathy syndrome, and are more frequently seen in AQP4+ than seronegative patients.^37–39^ MRI may also reveal long corticospinal tract lesions extending from the deep white matter in the cerebral hemisphere through the internal capsule to the cerebral peduncle.^36^ Lesions involving the corpus callosum are common in both NMOSD and MS, but can be distinguished by several features. In NMOSD, these lesions are often extensive and oriented along the long axis in a “bridge-arch,” and sometimes acutely edematous with a marbled pattern.^27,36,40^ In contrast, corpus callosum lesions in MS are characteristically well-circumscribed and ovoid-shaped, oriented perpendicular to the lateral ventricles (Dawson’s fingers).^41^ Brain lesions in NMOSD frequently decrease in size or resolve over time, whereas MS lesions rarely disappear.^42,43^ The enhancement pattern of brain lesions in NMOSD may be distinct with pencil-thin linear periependymal or “cloud-like” and poorly marginated enhancement.^27,44^ Leptomeningeal enhancement has also been reported.^36,45^ This is in contrast to the open-ring or nodular well-demarcated enhancement often seen in MS lesions.^46^

Another feature which may help differentiate brain lesions of MS from NMOSD is the “central vein sign.”^47^ With recent advances in imaging techniques, the “central vein sign” has emerged as a relatively specific radiological feature of MS, with studies showing that MS lesions form around central veins and venules in more than 80% of patients, as visualized on 3 T or higher-field strength MRI platforms.^48–51^ A study using a 3 T MRI scanner to differentiate AQP4+ NMOSD from MS found that the central vein sign was present in only 32% of NMOSD brain lesions patients compared with 80% of MS lesions (*p* < 0.001).^47^ Similarly, cortical lesions are frequently observed in MS and rarely occur in NMOSD and other MS mimics.^52,53^ These findings further underscore the differences in pathophysiology of these demyelinating diseases.

Discriminating between NMOSD and MOGAD using MRI brain is substantially more challenging.^32^ Like NMOSD, brain MRIs in MOGAD patients frequently display no lesions and are seemingly normal, with most of the abnormalities located on orbital MRIs.^33,54,55^ When abnormalities on brain MRI are found, they often consist of multifocal “fluffy” T2 hyperintensities in the deep gray or white matter.^56^ Compared with NMOSD, MOGAD patients more frequently have thalamic and cortical/juxtacortical lesions, the latter of which may be associated with seizures and encephalitis.^33,54,57–59^ Infratentorial lesions are typically more diffuse and more frequently involve the pons and middle and superior cerebellar peduncles in MOGAD, while lesions in the area postrema and medulla oblongata are rare compared with NMOSD.^33,54,55^

### Optic nerve lesions

Optic nerve lesions in NMOSD have distinct characteristics. Clinically, optic neuritis in NMOSD differs from MS in that it is more often the initial manifestation of the disease and produces more severe vision loss. Optic nerve lesions in NMOSD are often extensive, spanning greater than half of the length of the optic nerve with associated enhancement in the acute setting.^60–62^ They more commonly involve the posterior optic nerve, extending into the optic chiasm. MS optic nerve lesions, on the other hand, are usually shorter, more anterior, and unilateral.^46^ MRI features of the optic nerve may also be useful in differentiating NMOSD from MOGAD, in which optic neuritis is the most common clinical manifestation and is often recurrent and bilateral with pronounced optic nerve head swelling.^62^ Although lesions in MOGAD similarly often span greater than half the length of the optic nerve, they tend to be more anterior in location.^63^ Furthermore, characteristic perineural enhancement of the optic nerve sheath and peribulbar structures can distinguish MOGAD from its demyelinating counterparts.^64^

### Spinal cord lesions

**Figure 2.**
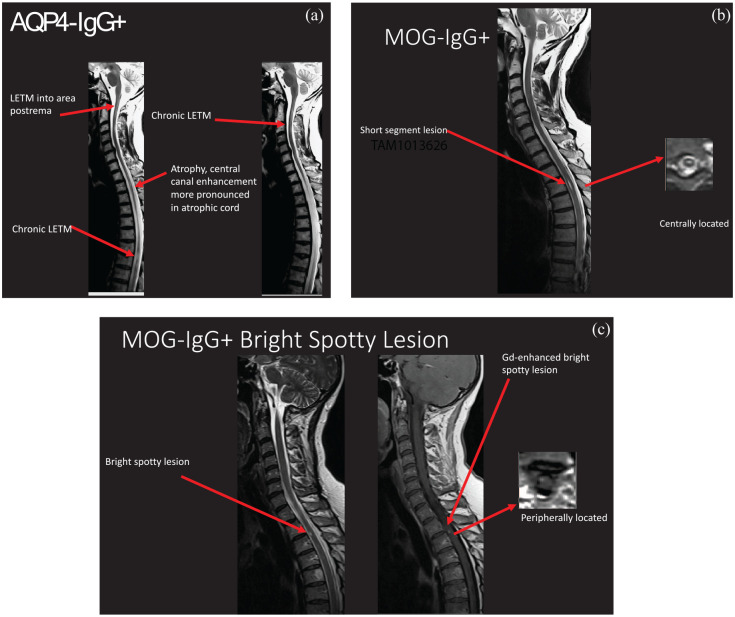
Imaging characteristics of spinal cord lesions in AQP4+ NMOSD and MOG+ myelitis. (a) AQP4-IgG+ disease, sagittal T2 sequences: longitudinally extensive lesions, extension of cervical cord lesions rostrally into the dorsal medulla, and subsequent cord atrophy and discontinuity of lesions in chronic LETM. (b) MOG-IgG+ disease, sagittal and axial T2 sequences: short-segment spinal cord lesions as demonstrated, LETM may also occur. (c) MOG-IgG+ disease, sagittal T2 and sagittal and axial T1 gadolinium-enhanced sequences: although bright spotty lesions are more frequently associated with AQP4-IgG+ NMOSD, they may rarely occur with other causes of transverse myelitis, as seen here. The short length and peripheral location of this lesion, in addition to serologic testing, helped to confirm the diagnosis of MOG-IgG+ disease. LETM, longitudinally extensive transverse myelitis; AQP4, aquaporin-4; MOG, myelin oligodendrocyte glycoprotein.

Spinal cord relapses in NMOSD usually cause a LETM, with the spinal cord lesion spanning three or more consecutive vertebral segments (Figure 2).^65^ However, up to 20% of patients may present with a short-segment transverse myelitis (STM).^66–68^ In these cases, one study found that 92% of subsequent myelitis relapses were LETM.^66^ Spinal cord lesions in NMOSD predominantly affect the central gray matter with associated hypointensity on T1-weighted MRI sequences. On axial images, spinal cord lesions may appear central or can occupy the full cross-sectional area of the spinal cord; the latter may arise owing to the considerable edema often arising from acute events. Lesions are rarely observed peripherally in the cord. Cervical spinal cord lesions may extend to the dorsal medulla. The enhancement pattern of spinal cord lesions in NMOSD is often patchy, though it can be diffuse or ring- or lens-shaped. In contrast, spinal cord MRI abnormalities in MS often consist of multiple lesions that are well demarcated and peripherally located in the posterior or lateral columns, and frequently occupy portions of both the gray and white matter.^46,69^ Furthermore, LETM is exceedingly rare in MS, though multiple short contiguous lesions in the spinal cord may mimic a longitudinally extensive lesion on sagittal imaging, emphasizing the importance of thorough review of axial sequences.^70,71^

Although the spinal cord lesions in NMOSD are typically longitudinally extensive, caution must be exercised when reviewing the MRI, as spinal cord lesions often evolve over time. Timing of imaging is crucial, since it may impact the length of the lesion and consequently the time to diagnosis and management of the disease.^66^ Although short-segment spinal cord lesions may lengthen over time, medullary lesions can extend caudally into the cervical spinal cord during a relapse.^72^ Therefore, if imaged too early, a longitudinally extensive spinal cord lesion may be missed. However, spinal cord lesions in NMOSD may continue to evolve after treatment of a relapse and during remission. Spinal cord lesions that were initially longitudinally extensive may become discontinuous over time, with several distinct shorter lesions seen on imaging.^73,74^ Eventually, and particularly with recurrent myelitis relapses, the spinal cord lesion may completely resolve and in some cases is replaced by spinal cord atrophy.^75^ Thus, late imaging may reveal short spinal cord lesions or no lesion at all, making diagnosis of LETM and NMOSD more challenging. Follow-up imaging with MRI of the spinal cord 6–12 months after a relapse can be useful to determine how the lesion has evolved, and may help differentiate a new lesion from an old one if another attack occurs in a similar distribution. Future studies may explore final spinal cord lesion length and development of spinal cord atrophy following a myelitis relapse as predictors of long-term disability outcomes.

Although LETM is common in NMOSD and is included in the diagnostic criteria of NMOSD, it is not specific to this disorder.^25^ Thus, when evaluating a patient with LETM, consideration must be given to other etiologies. Other causes of LETM include infection and parainfection, inflammation related to systemic autoimmune disease, granulomatous disorders, autoimmune glial fibrillary acidic protein (GFAP) astrocytopathy, spondylotic myelopathy, ischemia, metabolic disorders (deficiency in vitamin B12, copper, folate or vitamin E), neoplasm (most commonly astrocytoma and ependymoma) and paraneoplastic myelopathy, and spinal dural arteriovenous fistula.^76^ Describing the features of each is not within the scope of this article, but in the following we focus on several differential diagnoses that are more frequently encountered in clinical practice because of their similar clinical presentation to NMOSD (Table 1). LETM may be the initial clinical presentation of sarcoidosis, which may be initially misdiagnosed as NMOSD. Differing enhancement patterns may provide a clue in distinguishing the two disorders: one study found ring enhancement to be more common in NMOSD, while dorsal subpial enhancement and persistent enhancement for more than two months after corticosteroid treatment was more typical of sarcoidosis.^77^ Subpial enhancement together with central canal enhancement form the characteristic “trident sign” on axial images in neurosarcoidosis.^71^ Leptomeningeal and nerve root enhancement also favors spinal cord sarcoidosis, although nerve root enhancement has been described recently in select cases of MOGAD.^78^ In MOGAD, spinal cord lesions may be challenging to differentiate from NMOSD, since they are also commonly longitudinally extensive (though STM and multiple discrete lesions are not infrequent).^71,79,80^ However, their predominantly caudal location and more frequent involvement of the conus medullaris compared with NMOSD are helpful distinguishing features.^81^ Lesion enhancement is often patchy, and pseudo-dilation of the ependymal canal is a characteristic feature, while spinal cord atrophy is rare.^20,80–82^ Autoimmune GFAP astrocytopathy is a recently described autoimmune central nervous system (CNS) disease that may present with meningoencephalomyelitis and is characterized by the detection of GFAP-IgG antibody in the cerebrosinal fluid (CSF) and a perivascular radial enhancement pattern perpendicular to the lateral ventricles on brain MRI.^83^ Spinal cord lesions are usually longitudinally extensive, centrally located and involve the gray matter, but are more subtle with poorly defined margins and less edema compared with NMOSD lesions.^84,85^ Lesions often have linear central canal, punctate or leptomeningeal enhancement and the MRI abnormalities frequently recede after corticosteroid treatment.^85,86^ Other causes of LETM include spinal cord infarction, which is often confined to the anterior gray matter (“owl eyes”) with corresponding distribution of linear enhancement on sagittal sequences, and spondylitic compressive myelopathy, which has a predominantly central lesion and may demonstrate disc-shaped enhancement pattern (“pancake sign”).^71^ More recently, bright spotty lesions (BSLs), which appear as spotty lesions on axial T2-weighted imaging that are more hyperintense or of equivalent intensity to the surrounding cerebrospinal fluid, have emerged as a relatively specific radiologic marker for NMOSD. BSLs may facilitate the discrimination of NMOSD from other LETM etiologies (reported specificity up to 98.5% and positive predictive value up to 86.1% in anti-AQP4 seropositive patients).^87,88^ However, rarely BSLs can occur with other causes of transverse myelitis, including cases of MS^89^ and MOGAD (Figure 2c).^20^ Thus it is imperative to consider all features on spinal cord MRI, including the lesion length on sagittal sequences and central *versus* peripheral localization on axial images.

**Table 1.** MRI characteristics of NMOSD spinal cord lesions compared with other neuroinflammatory disorders.

	NMOSD	MS *versus* NMOSD	MOGAD *versus* NMOSD	Spinal sarcoidosis *versus* NMOSD	Autoimmune GFAP astrocytopathy *versus* NMOSD
Lesion length	Typically ⩾3 contiguous vertebral segments, though up to 20% are short	<3 contiguous vertebral segments, often multiple	<3 contiguous vertebral segments more common than in NMOSD	Typically ⩾3 contiguous vertebral segments, though may be short with tumefactive appearance	Typically ⩾3 contiguous vertebral segments
Location	Cervical and upper thoracic cord, predominantly central gray matter, often involve ⩾50% of the spinal cord axial cross-sectional area, may extend rostrally to the dorsal medulla	Cervical cord preference, peripheral in the posterior or lateral white matter	More caudal, may involve the conus medullaris	Cervical cord > thoracic cord, often dorsal	Often centrally located and involve the gray matter
Other characteristic imaging findings	Bright spotty lesions on T2 sequence, T1 hypointensity in acute lesions, spinal cord edema	Well-demarcated, asymmetric, T1 hypointensity rare	Pseudo-dilation of the ependymal canal with H-shaped hyperintensity seen on axial T2 sequences	Spinal cord edema	Lesions are more subtle with poorly defined margins and less edema
Characteristic symptoms	Para- or quadriparesis, sensory impairment, paroxysmal tonic spasms, bladder dysfunction	Sensory > motor impairment, Lhermitte’s sign, sphincteric symptoms	Flaccid paralysis, often prominent bowel, bladder, and erectile dysfunction; pediatric patients may have concurrent ADEM	Subacute onset of sensorimotor impairment, radiculopathy, bladder and bowel dysfunction	Sensorimotor impairment, often accompanied by meningoencephalitis
Enhancement patterns	Often patchy, but may be diffuse, ring- or lens-shaped enhancement sometimes seen	Nodular or open-ring enhancement	Patchy, may not enhance even when acute, pencil-thin linear enhancement of ependymal canal may be seen	Dorsal subpial, leptomeningeal or nerve root enhancement, “trident sign,” persistent enhancement >2 months after corticosteroid treatment	May have linear-appearing central canal, punctate or leptomeningeal enhancement on spinal cord MRI and perivascular radial enhancement perpendicular to ventricles on brain MRI
Post-attack imaging	May evolve into short distinct lesions or regress and be replaced by spinal cord atrophy	Complete lesion resolution may occur, spinal cord atrophy rare	Lesion resolution and spinal cord atrophy rare	Enhancement may persist	MRI abnormalities often resolve with corticosteroid treatment, but may become more prominent with dose reduction

ADEM, acute disseminated encephalomyelitis; GFAP, glial fibrillary acidic protein; MOGAD, myelin oligodendrocyte glycoprotein antibody disease; MS, multiple sclerosis; NMOSD, neuromyelitis optica spectrum disorder.

Recently, five validated imaging predictors for the differentiation of NMOSD and MS, named Cacciaguerra’s criteria, were described.^90^ These imaging criteria included the absence of combined juxtacortical/cortical lesions, the absence of ovoid periventricular lesions, the absence of Dawson’s fingers, the presence of LETM, and the presence of periependymal lesions along the lateral ventricles. Fulfillment of at least two of the five criteria distinguished NMOSD from MS with 92% sensitivity and 91% specificity in training samples, and 82% sensitivity and 91% specificity in validation samples.^90^ Cacciaguerra’s criteria, originally validated in a European cohort, have since been applied in a Chinese population to study their utility in a non-white population.^91^ Using a threshold of at least 3/5 criteria to distinguish NMOSD from MS, accuracy was 92%, sensitivity 91%, and specificity 93%. The criteria, however, failed to differentiate NMOSD from MOGAD. Distinct radiological diagnostic criteria for distinguishing NMOSD from MOGAD are therefore needed.

Children with AQP4+ NMOSD generally have similar imaging features to adults with this disease, but there are unique challenges in distinguishing NMOSD from possible mimics.^92^ Children with NMOSD are reported to more commonly have brain lesions and these may have a poorly demarcated, “fluffy” appearance.^32^ The presence of thalamic and internal capsule lesions, which are seen in ADEM but are infrequent in NMOSD, may help to distinguish NMOSD from this important differential diagnosis in childhood.^93^ LETM is a common feature on spinal cord MRI, but may more often be caused by other neuroinflammatory conditions including MOGAD and monophasic transverse myelitis.^92^

## Prognostication

The predictive value of various imaging markers for future relapses and clinical disability in MS is well established.^94,95^ In NMOSD, the utility of MRI for prognostication of disease course is less clear and studies are scarce (Table 2). However, prognostication at initial presentation of NMOSD is becoming increasingly important with the expansion in treatment options and resultant opportunity to individualize treatment strategies. With a lack of biomarkers to help predict disease course, MRI may serve as a useful tool and guide both acute and maintenance therapy selection.

**Table 2.** MRI features for prognostication in AQP4+ NMOSD.

Feature	Association	References
Spinal cord atrophy	Worse disability if atrophy observed at study baseline Multiple prospective and retrospective observational studies *Conflicting evidence about whether atrophy can occur in the absence of spinal cord relapses and visible lesions*	Chien *et al. Mult. Scler.* 2019 Liu *et al. Neurology.* 2015 Chien *et al. Brain communications.* 2019 Ventura *et al. Neurol Neuroimmunol Neuroinflamm.* 2016 Cacciaguerra *et al. Radiology.* 2020 Nakamura; *Eur. J. Neurol.* 2020
Spinal cord lesion length	Worse disability with longer acute spinal cord lesion length at time of attack Better recovery with STM but earlier and more frequent relapses Multiple retrospective observational studies	Mealy *et al. Mult. Scler. Relat. Disord.* 2019 Murchson *et al. J. Neurol. Sci.* 2015 Hu *et al. Mult. Scler. Relat. Disord.* 2018 (included some seronegative subjects) Jia *et al. Zhonghua Nei Ke Za Zhi.* 2015
Optic nerve lesion length	Worse long-term visual acuity with longer optic nerve lesion length One retrospective observational study	Akaishi *et al. J. Neuroimmunol.* 2016
Presence and evolution of acute brain lesions	Presence of acute symptomatic brain lesions at time of myelitis attacks associated with worse long-term disability; one multicenter retrospective study Better recovery with radiological resolution or decrease in brain lesion size, worse recovery with T1-hypointense lesions or cystic changes; one retrospective observational study *Conflicting evidence regarding whether presence of brain lesions predicts cognitive outcomes in NMOSD; May depend on focal brain lesions in thalamus*	Mealy *et al. Mult. Scler. Relat. Disord.* 2019 Kim *et al. PLoS One.* 2014 Cao G *et al.* Mult Scler Relat Disord 2020 Hyun J-W *et al.* Eur J Nerol 2017

NMOSD, neuromyelitis optica spectrum disorder; STM, short-segment transverse myelitis.

Several studies have evaluated the association between spinal cord atrophy and long-term disease outcome. Mean upper cervical cross-sectional area (MUCCA) at study baseline, utilized as a measure of spinal cord atrophy, has been shown to be associated with an increased number of myelitis relapses.^20,96,97^ In other studies, MUCCA at study baseline was predictive of subsequent clinical disability, including Expanded Disability Status Scale (EDSS), timed 25-foot walk speed, and 9-hole peg test.^13,20–22,97^ Interestingly, the association between MUCCA and disability has been found even in patients with NMOSD without a clinical history of myelitis or any spinal cord lesions, suggesting that subclinical spinal cord pathology may develop in NMOSD patients who have never had a clinical myelitis attack.^13^ However, there is conflicting evidence on this point, as a more recent study reported no evidence of spinal cord atrophy in the absence of cord lesions.^21^

In addition to spinal cord atrophy, spinal cord lesion length may have predictive value. In several studies, longer spinal cord lesion length at time of attack was associated with increased disability, both at attack-nadir and after recovery.^23,98^ Studies comparing NMOSD patients with LETM and STM have demonstrated that patients with STM have a better prognosis, with less motor and bowel or bladder disability, lower EDSS scores at nadir of first myelitis attack, and better recovery. On the other hand, patients with STM tend to relapse earlier and more frequently.^99,100^

Similarly, optic nerve lesion length, particularly the length of the intra-orbit and canalicular segments, in the acute stage of optic neuritis has been shown to correlate with long-term visual acuity in NMOSD.^24^ An emerging, clinically relevant technique that can be used as a complementary early imaging tool in NMOSD is optical coherence tomography (OCT), which reconstructs the retinal layers with high resolution.^101^ Thinning of the retinal nerve fibre layer (RNFL) after an optic neuritis relapse is usually more profound in NMOSD compared with MS, and the severity of retinal injury measured by OCT has been found to correlate with worse vision-related quality of life.^102–105^

Although optic neuritis and myelitis in NMOSD often lead to poor clinical outcome, patients with relapses localizing to brain lesions may have better overall recovery, although this requires further study.^43^ Although data are limited, there is evidence to suggest that evolution of acute brain MRI lesions in patients with NMOSD may help predict clinical outcome after a brain relapse, with good clinical recovery often seen in patients with radiological resolution or decrease in size of their brain lesions.^42,43^ One retrospective study following 63 NMOSD patients with an acute brain symptomatic relapse showed that patients with higher number of T1-hypointense lesions or cystic changes on follow-up MRI brain (obtained at a median of 21 months after initial MRI) had relatively worse recovery from the relapse.^43^ This is possibly explained by the consequences of axonal loss these MRI changes are thought to represent.

To date, the long-term clinical significance of asymptomatic brain lesions is not well understood. One large multicenter study examined brain MRI characteristics in Chinese NMOSD patients and found that patients with brain lesions, most of which were non-specific white matter changes, had a trend toward decreased subcortical gray matter volume compared with those without brain lesions.^106^ Although this may imply that brain lesions contribute to the development of gray matter atrophy, the authors did not find a relationship between the presence of brain lesions and cognitive impairment. There was also no significant difference in brain volume, including gray matter volume, between cognitively impaired and cognitively intact groups. However, one previous study had found differences in the hippocampus and other deep gray matter structures,^107^ and another study found that decreased thalamic volume, were associated with cognitive impairment in NMOSD. Taken together, these studies suggest that focal rather than global brain atrophy may be a key contributor to cognitive decline in NMOSD.^108^

With regards to lesion location in the CNS, some patients with NMOSD have a predilection to relapse in the same location in subsequent attacks. This concept has been illustrated in patients with relapsing–remitting MS, suggesting that the initial clinical demyelinating event location may predict the location of future clinical relapse locations.^109^ A retrospective analysis of patients with NMOSD revealed increased likelihood of a second relapse occurring in the same location as the initial attack, regardless of whether the first relapse location was in the brain, brainstem, optic nerve, or spinal cord.^110^ As mentioned, attacks in certain locations, such as the spinal cord, are associated more frequently with severe, irreversible disability and may imply a need for early aggressive therapy to prevent a similar future attack.^111^

Recently, mathematical models have been applied to large, multi-national patient datasets for prognostication in NMOSD.^112^ In one study, a modeling framework was built to understand the factors that predict future relapses and disability.^112^ The onset attack was not a strong contributor of long-term disability, which was better predicted by recurrent relapses. However, only clinical information was collected and used in this model. The inclusion of MRI data from the initial relapse could potentially improve the predictive power of the model for NMOSD disease course and warrants future study.

## Detection of disease activity

Relapses in NMOSD have a tendency to recur in the same location as previous clinical events. Furthermore NMOSD attacks can cluster, with multiple new relapses occurring within 12 months of each other.^113^ The close temporal relationship of relapses to each other coupled with a predilection to relapse in the same neuroanatomic distribution can make it challenging to distinguish a relapse from pseudorelapse without use of a gadolinium-enhanced MRI. A pseudorelapse can be defined as worsening in pre-existing neurologic symptoms due to a systemic insult, without evidence of new inflammatory disease activity.^114^ Often a clinical illness such as an infection triggers a pseudorelapse, but this is not always readily identifiable. The distinction of relapses and pseudorelapses is imperative, as it guides both acute and chronic treatment and avoids treatment delays and inappropriate use of immunosuppressive therapy. A new or enhancing lesion on MRI can help to confirm a true relapse, although in optic neuritis even enhanced orbital MRI sequences may occasionally miss a new lesion. A retrospective analysis of NMOSD patients hospitalized for presumed relapse illustrated that worsening visual symptoms and visual acuity could accurately distinguish a true optic neuritis relapse from a pseudorelapse.^114^ On the other hand, worsening motor dysfunction, sensory loss, and bowel and/or bladder symptoms could not reliably differentiate between a transverse myelitis relapse and pseudorelapse. In these patients, MRI of the spinal cord with contrast is a very helpful tool, with a new or enhancing lesion seen in most true relapses. There may be occasional episodes of myelitis where imaging is negative as has been reported recently with MOGAD.^115^ Thus a negative MRI does not completely rule out the possibility of a relapse, and further, ideally prospective, imaging studies are required to validate the sensitivity and specificity of MRI findings for the adjudication of relapses.

MRI has also proven useful as an adjunct tool for informed adjudication of relapses in the NMOSD clinical trials N-MOmentum, PREVENT, SAkuraSky, SAkuraStar, and TANGO.^8–12^ In most of these trials, MRI was not required for diagnosis of a relapse, but could be used as supportive evidence when relapses could not be adjudicated based on clinical criteria alone. In the N-MOmentum trial, MRI was required to meet protocol-defined relapse criteria in certain circumstances, and in practice, it was utilized in 37% of adjudicated relapses.^116,117^ As well, 16% of the cases deemed by the investigator to meet attack criteria were eventually rejected by the adjudication committee, with 75% of these rejections due to lack of new MRI findings indicating a relapse. These trials have provided a crucial learning experience, by illuminating the subjectivity of clinical criteria for relapses in NMOSD and utility of MRI to evaluate relapses and guide treatment decisions.

Recurring relapses in the same location in a patient with severe pre-existing disability from a previous clinical attack may be difficult to identify. For example, a patient with severe vision loss from a prior optic neuritis may not report visual changes in the same eye, and a clinical evaluation may not be sensitive enough to detect worsening. In these cases, MRI showing new enhancement may serve as a more sensitive marker to detect a relapse. This result could lead to further clinical exploration such as a dedicated ophthalmologic examination with high-contrast visual acuity or visual field testing. Verification of a change on exam, however subtle, may provide an opportunity to optimize disease control with a change in therapeutics. The presence of gadolinium-enhancing lesions in the absence of a clinical attack was recently reported to occur in approximately one-third of NMOSD cases in an abstract based on a *post hoc* analysis from the N-MOmentum trial, as yet unpublished.^118^ Most frequently, asymptomatic enhancing lesions occurred in the optic nerves but they were also observed in the spinal cord, and rarely, in the brain. The clinical significance of asymptomatic gadolinium-enhancing lesions in NMOSD patients remains unknown. Longitudinal study of routine MRIs with gadolinium in NMOSD patients will be necessary to clarify the prognostic value of these lesions over time.

In patients with acute myelitis or optic neuritis, concurrent brain lesions may not produce distinguishable clinical manifestations and might be overlooked. A retrospective study found that 15% of patients with a myelitis relapse and 8% of patients with optic neuritis relapse had acute asymptomatic brain abnormalities typical of NMOSD.^119^ Edematous corpus callosum lesions represented the most common asymptomatic brain lesions, followed by internal capsule and/or cerebral peduncle lesions. Interestingly, the authors found that the median time to diagnosis of NMOSD using the 2015 diagnostic criteria could be significantly shortened from 28 to 6 months if asymptomatic NMOSD brain lesions were included when determining dissemination in space. Other studies have similarly found that asymptomatic brain MRI abnormalities are common and may be more frequent than clinical symptoms localizing to the brain.^30,31,36^ However, some of these lesions consist of small or patchy non-specific T2-hyperintensities in the subcortical or deep white matter and are of uncertain significance. A recent study using serial MRI scans to investigate the occurrence of new, asymptomatic brain lesions in NMOSD patients over at least one relapse-free year found that only 3.4% of patients developed new and silent brain lesions during a total observed relapse-free period of 708 person-years.^120^ All the lesions detected were of non-specific appearance and location in the deep white matter. Similar findings have been observed in other studies.^121^ Dedicated longitudinal studies will be required to clarify the importance of asymptomatic brain lesions over time with respect to clinical outcomes such as cognitive status.

Though rare, asymptomatic enhancing and non-enhancing spinal cord lesions have been reported in patients with NMOSD without symptoms or signs of myelitis.^14,118,122^ In the reported cases outside of N-MOmentum, spinal cord lesions were all short, suggesting that STM is more likely to be asymptomatic than LETM. It is not yet clear how subclinical activity correlates with future relapses and disease course, and future studies addressing this gap are necessary.^13,15,16,123–125^

## Should MRI inform treatment decision-making in NMOSD?

In the last few years, the therapeutic landscape of NMOSD has changed dramatically with the approval of targeted, highly effective immunotherapies for NMOSD.^8,9,12^ The role of longitudinal routine MRI monitoring in NMOSD has not been established, but mounting evidence of subclinical tissue injury raises the question of whether regular follow-up imaging of NMOSD patients can inform treatment decisions in clinical practice.^13,14,16,119,126^ MRI monitoring of the brain and spinal cord could potentially help treating clinicians identify subclinical inflammation and allow them time to revise the treatment strategy before inflammation progresses to a full attack. However, one of the challenges of routine imaging is that inflammation leading to a clinical relapse may develop rapidly, and unless timed well, MRI may not capture evolving inflammation early enough to prevent its progression. As a result, it may be reasonable to consider pairing MRI monitoring with the use of a screening fluid biomarker. Several studies have explored the clinical utility of various fluid biomarkers in NMOSD, including GFAP and NfL, which are markers of astrocyte and axonal injury, respectively.^127–132^ One recent retrospective study demonstrated that serum levels of GFAP and NfL in NMOSD not only strongly correlated with CSF levels, but were also higher in NMOSD patients than in MS patients and healthy controls.^127^ As well, both serum GFAP and NfL levels have been shown to increase after recent relapses and correlate with EDSS scores in NMOSD.^127,132,133^ Further longitudinal study is warranted to determine whether biomarkers such as serum GFAP or NfL may rise in advance of clinical symptoms of an attack. If this were confirmed, then a fluid biomarker could be used as an initial screening step, followed by an enhanced MRI study, to screen for new inflammatory activity.

Various advanced imaging modalities are being investigated as tools to help quantify subclinical tissue damage in the brain, optic nerves, and spinal cord of NMOSD patients. If validated against clinical outcomes and accessible by most centers, these techniques may inform treatment decisions in the future. Studies using advanced MRI techniques in NMOSD are scarce and limited in their sample sizes, but demonstrate that advanced imaging is able to detect occult damage in the gray and white matter that is normal-appearing on conventional MRI studies.^124,134,135^ The extent of gray matter damage is controversial, as early data from diffusion-weighted imaging and brain tissue volumetry studies suggest greater white matter than gray matter degradation in the brain, while other studies have shown more tissue damage to the gray matter.^135–137^ Diffusion tensor imaging is another advanced MRI technique that has been used to evaluate microstructural changes in the normal-appearing white matter in NMOSD and has demonstrated that the white matter injury in NMOSD is diffuse, compared with MS in which the white matter alterations are predominantly periventricular.^16,138^ In addition, microstructural white matter changes have been identified in the afferent visual pathway in NMOSD patients with a history of optic neuritis, as well as in NMOSD patients with a history of LETM without optic neuritis.^16,139^ Diffusion tensor imaging has also been used to explore the differences in microstructural parenchymal damage between NMOSD and MOGAD and has shown that, compared with healthy controls, it is more prominent in MOGAD patients than in NMOSD patients, while brain volume loss is more severe in NMOSD patients.^140^

A recent study used quantitative spinal cord MRI to compare spinal cord characteristics in NMOSD, MOGAD, and MS patients with myelitis.^141^ NMOSD patients were found to have a significant reduction in cervical and thoracic cord cross-sectional areas, cervical cord gray matter, magnetization transfer ratio, fractional anisotropy, and increased mean diffusivity compared with healthy subjects. In contrast, there was no significant difference in these measures between MOGAD patients and healthy controls. Using cord metrics alone, NMOSD myelitis could be differentiated from MOGAD myelitis. As well, an association was identified between lower mean cervical cord cross-sectional area and EDSS, and between spinothalamic tract fractional anisotropy and pain in NMOSD, MOGAD, and MS.

Several studies using resting-state and task-based functional MRI (fMRI) have shed light on the dynamic changes occurring in specific regions of the CNS in NMOSD.^142–146^ One resting-state fMRI study showed that while the normal global topological structure was preserved, there was decreased functional connectivity in the visual and sensorimotor networks, and a positive correlation between neuronal reorganization and EDSS scores was demonstrated.^142^ Other studies have illustrated that NMOSD patients with a history of optic neuritis undergo selective reorganization of the visual network with connectivity changes.^147,148^ The alterations in functional connectivity were shown to be associated with reduced visual and acuity and frequency of optic neuritis.^143^ The exceptional resolution that ultrahigh-field 7-T MRI provides could further characterize structural changes in the CNS of NMOSD patients on a submillimeter level. Studies have used 7-T MRI to differentiate between NMOSD and MS and have shown that most NMOSD patients have small, subcortical lesions without a central venule, and an absence of cortical pathology.^48,149^ Further larger-scale longitudinal studies with advanced MRI techniques are needed, and importantly, efforts to validate and incorporate these imaging advances in the clinical context.

## Conclusions

Will MRI prove an essential tool for NMOSD prognostication and disease monitoring as we enter a new era of highly effective therapy? Can it provide a window into the future by flagging subclinical disease activity and offering an opportunity to optimize therapy before a disabling relapse occurs? MRI has long played an important part in NMOSD diagnosis by differentiating NMOSD from possible mimics, and thus enabling early initiation of immunotherapy. Once a diagnosis has been made, MRI can provide prognostic information and aid in the prediction of future attacks and long-term disability. During the disease course, gadolinium-enhanced MRI is helpful in confirming relapses and distinguishing them from pseudorelapses for prompt and appropriate treatment. Finally, radiological surveillance can aid in detection of subclinical disease activity, but the clinical significance of such activity needs to be further studied. Accumulating evidence suggests that asymptomatic gadolinium enhancement, spinal cord atrophy, loss of retinal ganglion cells, and microstructural changes in the brain occur independent of relapses. Large multicenter longitudinal studies with MRI and concordant biomarker monitoring of NMOSD disease activity are necessary to clarify the importance of these changes over time. As well, continued investigation of advanced imaging techniques in NMOSD will contribute to insights into underlying pathophysiological mechanisms. These findings will allow us to optimize and personalize therapeutic approaches in NMOSD, with the ultimate goals of minimizing long-term disability and improving the lives of patients.
